# Depth as an Organizing Force in *Pocillopora damicornis*: Intra-Reef Genetic Architecture

**DOI:** 10.1371/journal.pone.0122127

**Published:** 2015-03-25

**Authors:** Kelvin D. Gorospe, Stephen A. Karl

**Affiliations:** Hawai‘i Institute of Marine Biology, University of Hawai‘i at Mānoa, P.O. Box 1346, Kāne‘ohe, Hawai‘i, United States of America; University of California Santa Cruz, UNITED STATES

## Abstract

Relative to terrestrial plants, and despite similarities in life history characteristics, the potential for corals to exhibit intra-reef local adaptation in the form of genetic differentiation along an environmental gradient has received little attention. The potential for natural selection to act on such small scales is likely increased by the ability of coral larval dispersal and settlement to be influenced by environmental cues. Here, we combine genetic, spatial, and environmental data for a single patch reef in Kāne‘ohe Bay, O‘ahu, Hawai‘i, USA in a landscape genetics framework to uncover environmental drivers of intra-reef genetic structuring. The genetic dataset consists of near-exhaustive sampling (*n* = 2352) of the coral, *Pocillopora damicornis* at our study site and six microsatellite genotypes. In addition, three environmental parameters – depth and two depth-independent temperature indices – were collected on a 4 m grid across 85 locations throughout the reef. We use ordinary kriging to spatially interpolate our environmental data and estimate the three environmental parameters for each colony. Partial Mantel tests indicate a significant correlation between genetic relatedness and depth while controlling for space. These results are also supported by multi-model inference. Furthermore, spatial Principle Component Analysis indicates a statistically significant genetic cline along a depth gradient. Binning the genetic dataset based on size-class revealed that the correlation between genetic relatedness and depth was significant for new recruits and increased for larger size classes, suggesting a possible role of larval habitat selection as well as selective mortality in structuring intra-reef genetic diversity. That both pre- and post-recruitment processes may be involved points to the adaptive role of larval habitat selection in increasing adult survival. The conservation importance of uncovering intra-reef patterns of genetic diversity is discussed.

## Introduction

Local adaptation occurs when populations become fine-tuned to their environment through the process of natural selection. Locally adapted genotypes, however, can be swamped out by the arrival of immigrants from populations differently adapted. This is the dominant paradigm in terrestrial systems, where physical isolation is considered important, if not essential for populations to diverge [1]. In contrast, because many marine organisms begin their lives as planktonic larvae, the dominant paradigm in marine systems has been that the diversifying effects of natural selection would be homogenized by the high gene flow connecting populations in the ocean (discussed in [2]). This paradigm has now largely been abandoned, as numerous examples from fish [3, 4] and various marine invertebrates [5, 6] demonstrate that not only can marine populations become locally-adapted, but that ecological differences between populations can, in some cases, be strong enough to foster speciation events in sympatry [7].

In marine systems, indication of local adaptation comes from both experimental studies involving reciprocal transplant experiments (e.g., [8, 9]) as well as from statistical inferences based on correlating population genetic data with environmental variables (e.g., [10]) or in the detection of outlier loci that deviate from neutral expectations (e.g., [11]). Recently, we have uncovered both environmental [12] and genetic heterogeneity [13] in marine systems at small spatial scales (within a single patch reef of 40 m diameter) and local adaptation in marine systems has begun to receive increasing attention [3, 6, 14].

The term local adaptation implies the existence of patterns on a small spatial scale. This is often interpreted to mean adaptive differences between discreet demes existing in distinct habitats. It is important to note, however, that local adaptation also can refer to differences within continuous populations along a continuous environmental gradient [15]. Small-scale, local adaptation in plants often can be found in the form of genetic differentiation being structured along environmental gradients [16, 17]. This characteristic is believed to be common in plants because they are: (i) sessile, and thus more susceptible to small-scale environmental heterogeneity [18] and, in some cases, (ii) able to reproduce clonally, allowing selective factors to act on identical genotypes over multiple generations [19, 20]. Marine local adaptation has largely been viewed from a discreet deem or population standpoint [6], focusing on spatial scales of tens to thousands of kilometers. For example, while local adaptation has been demonstrated for corals on an inter-reef level, (e.g., [21, 22]), the potential for local adaptation on an intra-reef scale has rarely been investigated (but see [9]). Investigating patterns of small-scale genetic variation across a continuous landscape of environmental variation, as is done in landscape genetic analyses [23], may hold insight into the potential for natural selection to influence patterns of coral genetic diversity on an intra-reef scale.

Investigations that look at individual-level genetic variation across an environmental gradient are common in the terrestrial plant literature (e.g., [24]). Corals share the same sessile and reproductive traits that predispose plants to local adaptation. Furthermore, it could be argued that corals may have an increased potential to exhibit genetic patterns along an environmental cline because unlike plants, the dispersing propagules of corals actively engage in site selection. The ability of larvae in sessile marine invertebrates to sense and be attracted to specific settlement cues has been shown to be intra-specifically variable and heritable [25–27] as well as resulting in an increased probability of survival [28, 29] indicating an evolutionary potential for natural selection to act on this trait. Indeed, a variety of biotic and abiotic settlement cues are known to act on a range of spatial scales from long-distance cues (e.g., open ocean currents) that influence larval transport towards islands to those that influence larval site selection within a reef (e.g., sedimentation) to still others (e.g., depth, temperature, light quality intensity, chemical cues) that likely influence larvae throughout their dispersing stage [30, 31]. Therefore, it is possible that adaptive genetic divergence in corals may be found on an intra-reef scale. It has long been recognized that reefs exhibit zonation patterns in the distribution of coral species [32] largely driven by environmental heterogeneity and inter-specific larval behavioral differences [29, 33], but it remains to be tested whether intra-specific, individual-level genetic variation also plays a role in the genetic architecture within a reef.

In a recent study [13], we genotyped and mapped nearly every individual of the coral, *Pocillopora damicornis*, within a single reef. The reef we chose was in Kāne‘ohe Bay, Hawai‘i, USA and was ∼40m in diameter with an abundance of *Pocillopora damicornis* colonies and a depth range of between 5 and <1 m. *P*. *damicornis* is distributed across the Indo-Pacific and can be quite common in some locations. In our genetic study [13], we found positive spatial autocorrelation at short distances (< 5 m) and suggested that this pattern was driven by larval dispersal processes, whereby both clonally and sexually produced planulae of *P*. *damicornis* tend to settle nearby their brooding parent. What remained speculative, however, were the causes of negative spatial autocorrelation in larger distance classes (> 15 m), where corals were more unrelated to each other than expected at random. Indeed, a major question remaining unanswered is to what extent are geographic patterns maintained by drift (i.e., restricted dispersal and spatial processes) *versus* environmental heterogeneity (i.e., natural selection and larval recruitment behavior). In other words, there is a need to disaggregate the contributions of spatial and environmental processes that underlie the observed pattern of genetic variation. By allowing for the control of spatial dependence in environmental variables, landscape genetic analyses move beyond simply describing patterns of genetic and environmental variation and attempt to elucidate the processes that are responsible for generating them [34, 35]. Here, we integrate our spatial and genetic datasets [13] with depth and temperature datasets collected for the same reef [12] to examine how environmental characteristics may be influencing intra-reef spatial genetic patterns. We determined that pre- and/or post-settlement processes are acting to create spatial patterns of coral genetic relatedness at an intra-reef scale.

## Methods

### Genetic, spatial, and size data collection

Coral sampling and microsatellite genotyping are described in a previously published study [13]. Briefly, a small ∼40 m diameter patch reef (Reef 19) in Kāne‘ohe Bay, O‘ahu, Hawai‘i, USA was selected and exhaustively surveyed for *P*. *damicornis*. Transect methods using benthic-distances were used to measure the spatial coordinates (x, y) of each individual (defined here as a single, discreet coral colony). Each coral individual was genotyped at six microsatellite loci (Dryad accession doi:10.5061/dryad.968k6). In addition, sequencing of a mtDNA open reading frame of unknown function ([36]; GenBank accession numbers KP698585-KP698587) and principal components analysis of the microsatellite dataset [13] indicated that the presence of cryptic species is unlikely. The image analysis software, *ImageJ* (ver. 1.45s; [37]) was used to measure the planar surface area (i.e., as projected on a 2D photograph) of each individual coral based on size-standardized photographs taken in the field. Finally, it should be noted that Hardy-Weinberg equilibrium and locus-level analyses as well as spatial and clonal structure analyses are highlighted elsewhere [13] such that, here, we focus on the landscape genetics analyses.

### Environmental data collection

High-Resolution Thermochron iButton temperature and time data loggers (model DS1921H; Maxim Integrated Products, Inc., Sunnyvale, CA, USA) were deployed on a 4 m grid throughout Reef 19 (85 locations). Data for this study are based on two years of temperature recordings collected between November 2007 and November 2009 and published previously [12]. In addition, depth measurements were taken at each of the 85 temperature monitoring stations and standardized by tide level.

Many characteristics of sea temperature are indeed correlated with depth (e.g., average daily temperature, average daily temperature range, average monthly temperature, etc.) and therefore, are not included in subsequent analyses to avoid collinearity between variables. Based on our previous study [12], however, we found two temperature indices could not be explained by depth based on partial regression analyses. The two temperature indices were Relative Hotspots and Relative Hothours [12] and are included in this study. The Relative Hotspots index is defined as the proportion of time over two years during which a location was one or more standard deviations hotter than the average temperature for the whole reef. The Relative Hothours index, on the other hand, is defined as the proportion of time spent over the course of two years during which the temperature at a location was one or more standard deviations hotter than the average temperature for that same location in the past twelve hours. Thus, areas of the reef that are Relative Hotspots are frequently warmer than spatially averaged temperatures for the entire reef, while areas of the reef that are Relative Hothours are frequently warmer than site-specific temporally averaged temperatures.

We then combine the Hotspots and Hothours indices information of the 85 temperature monitoring sites with their individual spatial locations to spatially interpolate a Hotspots and Hothours map for the entire reef. To do this, we use the gstat package [38] in R to perform ordinary kriging, a geostatistical spatial interpolation method that models the relationship between distance and variance of sampled points to predict values at unsampled locations. Our dataset of coral spatial coordinates was then layered on top of these maps to obtain an estimate of the Hotspot and Hothour Index for each coral colony. Lastly, the same statistical techniques were applied to our depth measurements to generate a bathymetric map of the reef [12] and estimate the depth at which each coral colony resides.

### Data analysis

First, we calculated pairwise Euclidean distances for each individual coral’s spatial coordinates and environmental data (i.e., depth as well as Hothours and Hotspots indices). For the genetic data, coefficients of relationship based on Moran’s *I* [39] were calculated using the program Spatial Pattern Analysis of Genetic Diversity (SPAGeDi; [40]) for all pairs of coral individuals. These five measures (i.e., physical distance, Hothours, Hotspots, depth, and relatedness) were then used for subsequent analyses investigating the relationship, if any, between genetic relatedness and one or a combination of the spatial or environmental variables.

In order to account for spatially autocorrelated environmental data, we performed separate partial Mantel tests [41, 42] to calculate the ranked correlation (i.e., analog to a Spearman’s correlation) between genetic relatedness and each of the three environmental variables (i.e., Hothours, Hotspots, depth), while controlling for the effect of spatial distances. The partial Mantel test calculates the correlation between two distance matrices, A and B, while controlling for the effect of a third, C, by calculating the correlation between the matrices of residuals between A and C and A and B. Furthermore, statistical significance is based on creating a null distribution by Monte Carlo randomization, whereby one of the matrices is unmanipulated and the other is randomly permuted [42]. While past attention has criticized the use of the permutation procedure for the partial Mantel test [43, 44], studies confirm that for sample sizes greater than ∼50, permutation procedures remain valid [45, 46]. In addition to treating depth as an environmental variable in which spatial autocorrelation must be controlled for, we also tested for the relationship between genetic relatedness and spatial distance, while controlling for the effect of depth. This allows us to tease out the influence that depth may have on spatial location and *vice versa*.

Furthermore, to investigate how the genetic correlation with space and each of the environmental variables may have changed over time, we repeated the partial Mantel test analyses on several subsets of coral samples after incorporating the size of the colony (i.e., planar surface area) as a proxy for age. Note that while issues of partial mortality, fragmentation and individual variation in growth rate make the correlation between area and age less than perfect in *P*. *damicornis*, it is still true that older corals tend to be larger than younger corals [47], thus allowing us to bin corals into various size classes. Corals with surface area < 10 cm^2^ (i.e., max diameter ∼3.5 cm) were placed into the smallest size class and are considered to be relatively recent recruits on the reef (i.e., less than 2 years old; [48]). Defining a bin size for our oldest size class, however, is complicated by the fact that colonies may suffer from fragmentation or may be the result of fusion between two originally separate colonies (i.e., larger size classes will likely have a larger variance in age; [47]). To reflect this uncertainty, we vary the lower bound of our largest size class and define the oldest recruits on the reef to be those with planar surface areas greater than 30, 40, 60, or 90 cm^2^ (i.e., the bins for older corals are overlapping). Thus, in addition to the partial Mantel tests performed on the entire dataset as described above, we also ran partial Mantel tests for each bin investigating the relationship between genetic relatedness and each environmental variable (while controlling for space) as well as between genetic relatedness and space (while controlling for depth). We also calculate pairwise genetic fixation indices (*F*_*ST*_) between our small size class and each of our large size class bins based on an analysis of molecular variance (Amova; [49]) as implemented in the program, Genodive [50]. For this analysis, we remove all repeated multi-locus genotypes (MLGs) within each size class bin and calculate significance based on 9999 permutations.

We used an information theoretic approach [51] to decide which environmental variables to interpret further by modeling genetic relatedness as a function of each variable described above (i.e., four bivariate analyses of genetic relatedness as a function of spatial, depth, Hothours, or Hotspots distances) as well as all possible linear combinations of them (i.e., 11 multivariate analyses of genetic relatedness as a function of two, three, or all four environmental variables). To do this, we use the package MuMIn [52] in R. Akaike weights (i.e., normalized likelihood values; *w*) were calculated for each model and all models were ranked based on Akaike’s information criterion (AIC; [53]). For each model, i, if AIC_i_—AIC_min_ > 5 this model was considered to be poorly supported and therefore not considered further [51]. Finally, for each predictor variable, the AIC weights are summed for all models containing that variable. A predictor weight (*w*_*+*_) is thus calculated for each variable, allowing the variables to be ranked in order of their importance. Thus, in contrast to simply selecting those variables that are contained in the single best model, inference is based on the entire set of models (i.e., multi-model inference; [51, 54]).

Overall, distance-based methods such as partial Mantel tests may be less powerful than methods based on raw data [46]. Furthermore, standard genetic differentiation analyses (i.e., *F*_*ST*_) also may be too coarse for the fine-spatial scale patterns in which we are interested. Thus, we perform a spatial principal components analysis (sPCA; [55]) using the Adegenet package in R [56]. Unlike PCA, which seeks new axes (i.e., principal components) to summarize the data based on maximizing the genetic variance among individuals, sPCA seeks new axes that optimize the product of genetic variance and their spatial autocorrelation as measured by Moran’s *I*. The calculation of Moran’s *I* requires that neighboring entities in the dataset be defined by a connection matrix. Here, we define neighbors as any set of coral individuals within a certain distance from one another (i.e., a neighbor by distance connection network). We use the program SPAGeDi to calculate the mean of the largest distance bin to exhibit positive autocorrelation and use this as the upper distance limit of neighbors. For this autocorrelation analysis, we use bins that create approximately equal numbers of pairwise comparisons per distance bin and we use 200 permutations of our spatial locations to define statistically significant autocorrelation.

Using both the connection matrix and a matrix of individual allele frequencies, sPCA results in both positive and negative eigenvalues, corresponding to positive (i.e., clines whereby neighbors tend to be genetically similar) and negative (i.e., patches whereby neighbors tend to be genetically different) spatial structures [57]. Each principal component axis represents a different spatial structure, and the decision of which axes to retain for interpretation is based on inspection of the scatter plot of all eigenvalues decomposed into their genetic variance and spatial autocorrelation components (i.e., screeplot). For visualization purposes, the mean PC scores of each individual’s neighbors (i.e., lagged or de-noisified scores) are then plotted back to that individual’s spatial coordinates to reveal the genetic spatial structure of the reef. To test for significance of global (i.e., positive) and local (i.e., negative) spatial structures, we use the Jombart et al. method [55] involving spatial filters created by decomposing the connection matrix into a set of Moran’s eigenvector maps (as is done in principal components of neighbor matrices; [58, 59], see [12] for an example). Statistically significant global structure would indicate the existence of a genetic cline (i.e., a pattern whereby nearby individuals tend to be more related than distant individuals) while statistically significant local structure would indicate the existence of genetic patches (i.e., a pattern whereby nearby individuals tend to be genetically different from their neighbors). The matrix of individual allele frequencies is then correlated separately for global and local filters and statistical significance based on a Monte Carlo randomization procedure in which we use 9999 matrix permutations to generate a null distribution. Here, the null hypothesis is that individual allele frequencies are randomly distributed throughout our connection network, while the alternative hypothesis is that individual allele frequencies display at least one global or local spatial structure [55].

### Ethics statement

*P*. *damicornis* is a protected species in Hawai‘i and all samples were collected under the Hawai‘i Institute of Marine Biology special activities permit approved by Department of Land and Natural Resources, Division of Aquatic Resources. Since *P*. *damicornis* is a non-vertebrate subject, no Institute Animal Care and Use Committee approval is necessary.

## Results

**Fig 1:**
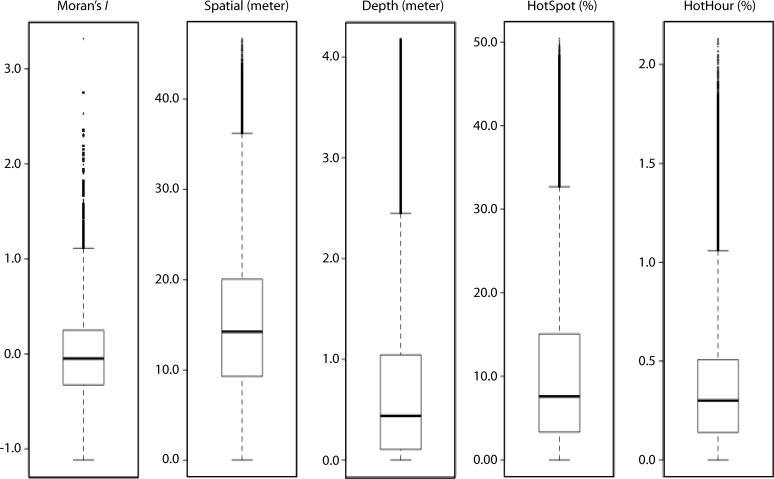
Boxplots of pairwise coefficients of genetic relationship as well as spatial and environmental distances. The rectangles represent the interquartile range [i.e., lower 25^th^ percentile, median (solid line), and upper 75^th^ percentile], the whiskers represent 1.5 times the interquartile range, and points represent outliers.

To highlight the small scale of our landscape genetic dataset, we provide boxplots (Fig. 1) of all pairwise coefficients of genetic relationship as well as all spatial and environmental variables. A total of 2352 individuals had complete six-loci genotypes as well as *in-situ* spatial coordinates and spatially-interpolated environmental data. Notice for all datasets, there is a preponderance of small distances between colonies with larger distances less common. For genetic relatedness (Moran’s *I*), this indicates a high frequency of closely related pairs of individuals, as was previously discussed in [13]. For spatial distances, this pattern of mostly small distances is expected for any sampling scheme as larger distances are confined to pairs of the relatively fewer outer sampling points (i.e., the number of individual separated by a certain distance decreases with increasing distance). For our environmental distances of depth, Hotspots, and Hothours, this simply is a reflection of normally-distributed data, with distances between points around the mean characterizing most of the small distances and distances between outlying points characterizing the relatively fewer larger distances.

The results of all simple and partial Mantel tests are shown in Tables 1 and 2. Here, a significant and negative Mantel *r* indicates a weak relationship between that variable and genetic relatedness, whereby closely related corals (large genetic relationship coefficients) tend to be found in similar environments (small environmental distance). All environmental variables (i.e., space, depth, Hothours and Hotspots) displayed this relationship under a simple Mantel test. When autocorrelation is taken into account with the partial Mantel test, however, only depth and space were found to be statistically significant (Table 1). It should be noted, however, that partial Mantel tests are based on pairwise distances and not raw data values, therefore, Mantel *r* values cannot be squared and interpreted as the percent of variance in the response variable explained by the predictor variable (i.e., the coefficient of determination; *r* ^*2*^) as is done in regression analyses [46]. For 2229 of the 2352 genotypes (94.8%), we had size data, which we binned into size classes. The results of partial Mantel tests comparing smaller (< 10 cm^2^) *versus* larger (> 30, 40, 60, or 90 cm^2^) corals are reported in Table 2. Once again, only depth and space were significantly correlated with genetic relatedness for smaller corals. For depth, the correlation with genetic relatedness increased for larger corals (−0.020 to −0.030) when compared to smaller corals (-0.014). In contrast, the correlation of spatial distance and genetic relatedness was only significant for the smallest size class. Lastly, based on our AMOVA, all estimates of genetic differentiation (*F*_*ST*_) between our small and each of our large size class bins were not significantly different from zero (0.948 < *p* < 1.000), indicating a lack of overall genetic differentiation between younger and older corals.

**Table 1:** Results of landscape genetic analyses comparing the relationship between genetic relatedness and each of four spatial or environmental predictor variables.

Variable	Mantel r (p-value)	Partial Mantel *r* (p-value)	*w*_*+*_
Depth	**−0.017** (0.001)	(controlling for space) −**0.011** (0.002)	1.00
Space	**−0.016** (0.001)	(controlling for depth) −**0.008** (0.001)	0.97
Hotspot Index	**−0.005** (0.031)	(controlling for space) −0.004 (0.07)	0.46
Hothour Index	**−0.006** (0.007)	(controlling for space) −0.002 (0.13)	0.30

Reported for each variable are Mantel *r* correlation coefficients from simple and partial Mantel tests and predictor weights (*w*_*+*_) from multi-model inference. Significant tests are only available for the simple and partial Mantel tests. Values with p-values < 0.05 are in bold.

**Table 2:** Partial Mantel *r* correlation coefficients between genetic relatedness and space (controlling for depth) and between genetic relatedness and each environmental variable (controlling for space) for different size class bins based on surface area.

Size	*N*	Depth	Space	Hotspots	Hothours
< 10 cm^2^	1037	−0.014**	−0.004*	−0.004	0.002
> 30 cm^2^	486	−0.020*	−0.005	−0.008	−0.004
> 40 cm^2^	370	−0.022*	−0.002	−0.013	−0.007
> 60 cm^2^	207	−0.012	0.005	−0.027*	0.009
> 90 cm^2^	95	−0.030*	0.026	−0.027	−0.004

*N* indicates the number of coral individuals (* *p* ≤0.05, ** *p* ≤0.01).

After model ranking based on AIC, only four models had AIC_i_—AIC_min_ < 5, and were thus selected for further consideration (Table 3). The model with the most support (i.e., lowest AIC) only contained depth and space as predictors of genetic relatedness. The AIC values for many of the models including Hotspots and/or Hothours, however, were relatively close (ΔAIC < 2) and thus hold considerable support of their own. Predictor weights calculated by multi-model inference (i.e., model averaging) for each environmental and spatial variable (*w*_*+*_) are reported in Table 1. Based on multi-model inference of predictor weights, only space and depth were highly supported variables.

**Table 3:** Model selection results on the response of genetic relatedness to all possible linear combinations of depth, space, Hotspots, and Hothours.

Landscape Model	*K*	log(L)	AIC	ΔAIC	*w*_*+*_
β_0_ + β_1_ (Depth) + β_2_ (Space)	4	−1832529	3665067	–	0.362
β_0_ + β_1_ (Depth) + β_2_ (Space) + β_3_ (Hotspots)	5	−1832529	3665067	0.27	0.317
β_0_ + β_1_ (Depth) + β_2_ (Space) + β_3_ (Hothours)	5	−1832529	3665068	1.64	0.159
β_0_ + β_1_ (Depth) + β_2_ (Space) + β_3_ (Hotspots) + β_4_ (Hothours)	6	−1832528	3665069	1.96	0.136

Only those models with ΔAIC = AIC_i_—AIC_min_ < 5 are shown. Number of parameters (*K*), log likelihood [log(L)], Akaike’s information criterion (AIC), and Akaike weights (*w*_*+*_) are reported for each model.

**Fig 2:**
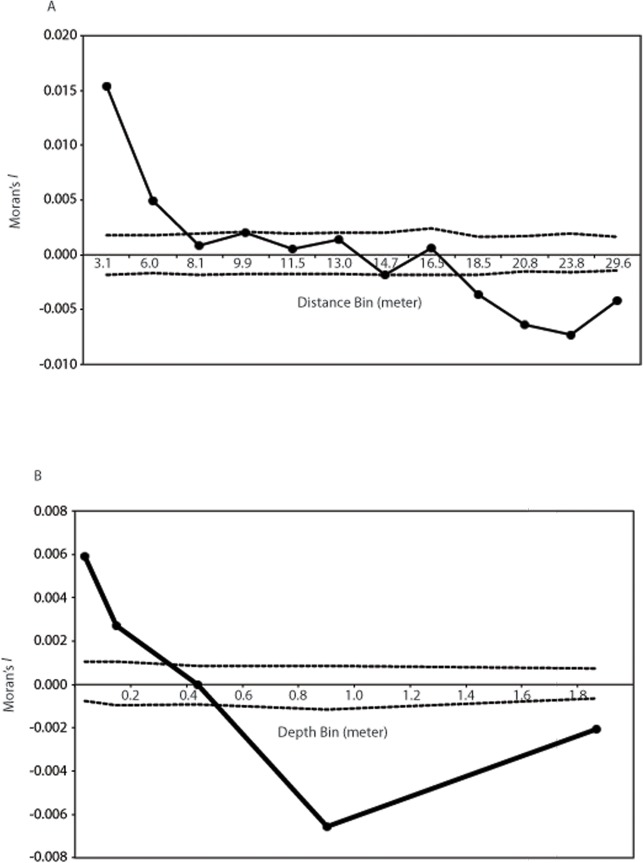
Results of spatial (A) and depth (B) autocorrelation analyses. In both analyses, the number of pairwise comparisons per bin was equalized. Dashed lines represent 95% confidence intervals based on 200 permutations of individual spatial coordinates or depth among all individuals.

In our sPCA analysis, since space and depth both appear to be significant drivers of genetic variation, we perform two separate analyses: one using each coral’s depth and another using spatial (x, y) coordinates. Thus, for our connection networks, we performed two separate spatial autocorrelation analyses comparing genetic relatedness with spatial distances (Fig. 2A) and genetic relatedness with depth distances (Fig. 2B) to define the upper distance limit of neighbors. Although theoretically possible, it should be noted that we do not perform a three dimensional sPCA using x, y, and depth coordinates because in this study we consider depth and space as separate, potential drivers of genetic variation. In our interpretation of sPCA, we consider depth-driven genetic variation as an environmentally-driven larval site selection process and space-driven genetic variation as a dispersal-limited process and, thus, analyze them separately.

**Fig 3:**
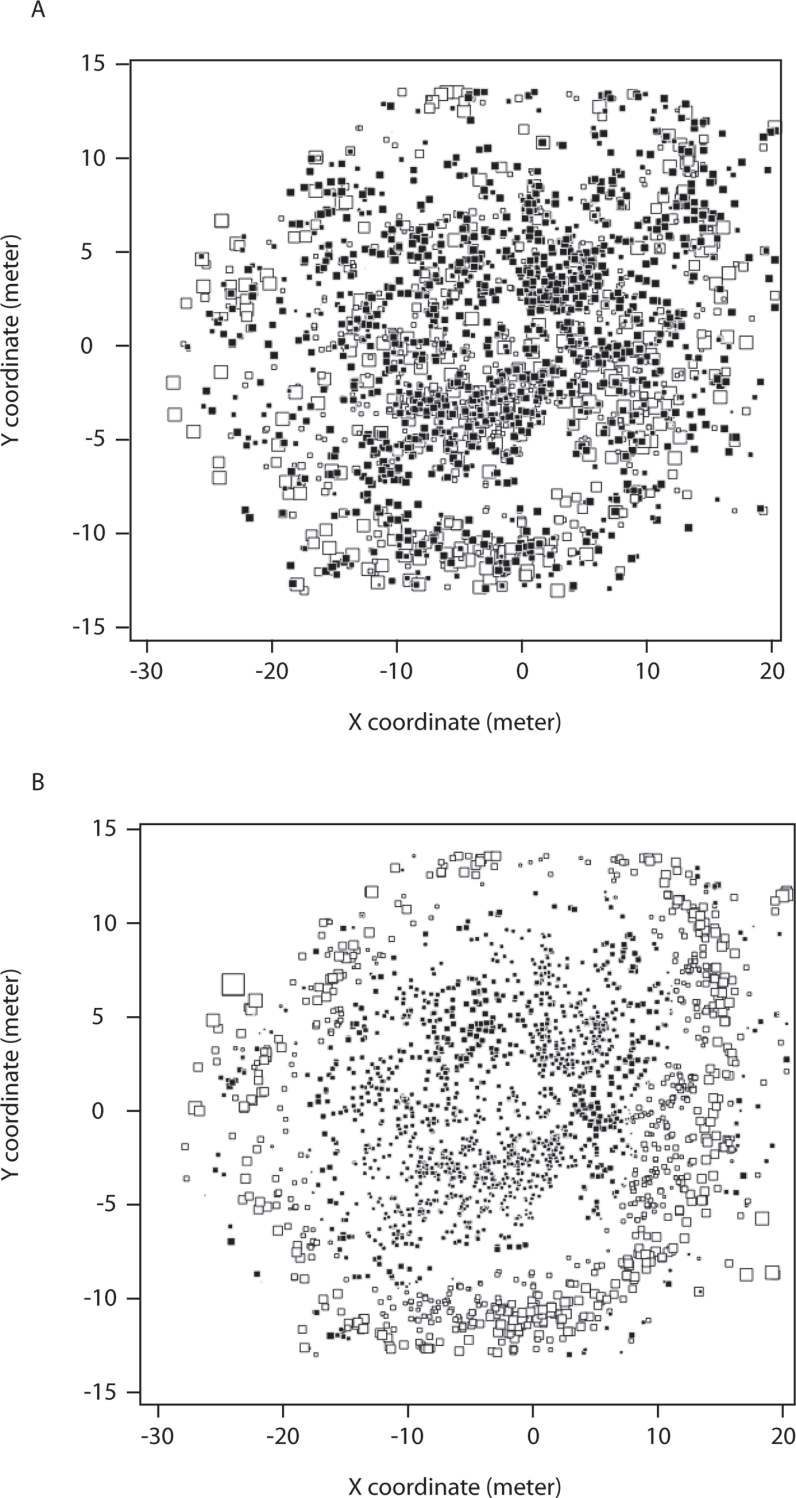
Spatial map of non-lagged (A) and lagged (B) scores from the first global principal component of sPCA based on depth. Each coral colony is represented by a box. The size of the box indicates the magnitude of the PC scores and filled boxes are positive and open boxes are negative values.

Based on the Monte Carlo randomization test, our sPCA using depth showed significant positive correlation (i.e., genetic cline; *p* = 0.019), while our sPCA using spatial coordinates did not (*p* = 0.102). Neither analysis, however, showed significant negative correlation (i.e., genetic patchiness; *p* = 0.936 for space and *p* = 0.840 for depth), and therefore we only consider the depth-based, positively correlated structures further. Based on the screeplot for our depth sPCA, we see that all global and local principal components contain relatively similar amounts of spatial autocorrelation, but only display results (Fig. 3) for the first global principal component (λ_1_), as this contained most of the genetic variance in the data. Mapping the PC scores of each individual back to their x, y coordinates revealed a depth cline in genetic variation with corals in the shallow, center portion of the reef and corals on the outer and deeper edges of the reef generally showing negative and positive PC scores, respectively (Fig. 3A). The pattern becomes even more pronounced when the lagged (i.e., de-noisified) PC scores are plotted (Fig. 3B). Global structures of other depth-based principal components exhibited similar spatial structures (not shown). For exploratory purposes, we also translate each of the first three global components of our sPCA based on spatial coordinates into a color intensity (red, green, and blue) to visualize them simultaneously (S1 Fig.). The resulting map displays what also appears to be a depth cline in genetic variation with colonies in the center portion of the reef appearing to have different combined PC scores from corals on the outer edges of the reef.

## Discussion

### Depth as a selective factor

Uncertainty in explaining patterns of genetic diversity can originate from multiple processes converging, particularly at small-scales (e.g., [60–63]). We have attempted to reduce this uncertainty through our intense characterization of our study site as well as through the use of spatially-explicit analyses. Depth and spatial location are correlated such that corals found at the same location will have predictable depths and *vice versa*. Furthermore, our relative Hotspots and Hothours temperature indices are correlated with space [12]. We tease apart the effects of depth, temperature and space using our partial Mantel tests, and find only depth and space to be significantly correlated with genetic relatedness. In addition, the results of our partial Mantel tests are corroborated by two other data analysis approaches: multi-model inference and sPCA. Since spatial processes were discussed previously [13], here we focus on the causes and consequences of genetic diversity patterned along a depth gradient. This study joins only a few [64–66] that have focused on the depth-associated distribution of the host coral’s genetics.

Based on the Monte Carlo randomization procedure of our sPCA, allele frequencies at our study site show positive autocorrelation (*p* = 0.019) with regards to depth. In other words, genetic diversity exhibits a depth cline whereby individuals at similar depths tend to have more similar genotypes than individuals at different depths. When we restrict our partial Mantel tests to the smallest size class, we see that this is true even for likely recent coral recruits. Planulae of *P*. *damicornis* have the ability to remain in the plankton for more than 100 days after release [67]. While this might point to the potential for *P*. *damicornis* to disperse over long distances, it has also been shown that planulae are competent (i.e., able to respond to settlement cues and begin metamorphosis) as soon as 12 h after release [68]. Given the weak, but significant correlations between genetic relatedness and depth distances (Tables 1 and 2), our data points to a detectable role for depth in larval habitat selection at this scale. That is, recruiting larvae may be selecting for a similar depth as their colony of origin.

In addition to genetics and depth being correlated for the smallest size class, we also find that the magnitude of this correlation increases when we only consider larger size classes (Table 2). Furthermore, based on our AMOVA, groups of corals in different size classes were genetically similar, thus ruling out the possibility that the temporal change in genetic-depth correlation could be due to genetic differences between recruiting cohorts (i.e., chance recruitment events). Put another way, since there are no genetic differences between newer and older coral recruits overall, the increasing correlation between genetic relatedness and depth for increasing size classes is being driven by differences in the depth distribution between older and younger corals, thus pointing to depth as a selective factor in post-recruitment processes. In particular, increased environmental dependence of genetic relatedness for larger size classes may be explained by selective mortality (e.g., [24, 69]). Indeed, pre- and post-recruitment processes are linked for marine larvae as metamorphosis is irreversible and behavioral selection of a settlement site could increase adult survival. While depth has been shown to influence larval swimming behavior [70], it is difficult to explain how depth alone (i.e., hydrostatic pressure) could play a selective role in structuring intra-reef genetic diversity.

It should be emphasized, that our Hotspot and Hothour temperature indices were specifically chosen for this analysis because they were independent of depth [12]. Our use of depth as an explanatory variable, however, may potentially be serving as a proxy for other temperature characteristics that are in fact depth-dependent. For corals, temperature appears to be an important environmental selective factor, with different populations exhibiting different bleaching responses [21, 71, 72], or stress protein expression levels [9] associated with different temperature regimes. The importance of temperature in adult survival, therefore, makes it a likely candidate as an important settlement cue.

On the other hand, another potential cause of depth-dependent genetic structuring to consider is the role of light in affecting coral larval settlement [73]. Selecting a habitat with a suitable light regime is important for coral adult survival, especially given the role of their intracellular, photosynthetic *Symbiodinium* spp. from which they derive most of their energy. Indeed, the marine environment experiences considerable spatio-temporal variation in spectral quality and light intensity [74]. This is particularly true for our study site, where for several hours during each of the extreme low spring tides, the top of the reef can find itself just a few centimeters from the surface of the water. More crucially than corals found in the deeper areas of the reef, corals in this extreme shallow environment must balance the need for photosynthetically-active light with the potential damages of increased ultraviolet light irradiance [75]. In fact, it also has been shown that larvae originating from deeper colonies have lower survivorship when exposed to light spectra more typical from shallow depths [76]. Thus, while our results indicate a correlation between genetic relatedness and depth, it is important to keep in mind that the proximal, causative factor responsible for these patterns may in fact be any number of depth-dependent environmental variables. Confirmation of the specific mechanism by which depth could influence patterns of genetic diversity on an intra-reef scale requires further exploration.

### Conservation implications

If the environmental heterogeneity found within a reef is enough to structure genetic diversity patterns along a depth cline then this has important conservation implications. Recall that the lagged PC scores (Fig. 3B) are obtained by averaging for each individual the scores of its neighbors as defined by the connection network. In this sense, our lagged sPCA map represents spatio-genetic variance averaged among neighbors throughout the reef, while our non-lagged sPCA map (Fig. 3A) represents the non-averaged, individual data. If the depth cline we observed at our study site is due to the selective factors discussed above, then a comparison of our non-lagged *versus* lagged maps could help to identify individuals whose PC scores grossly differ from their neighbors. If the selective factor is strong enough, then these individuals will eventually be weeded out by selective processes. This may even explain the occurrence of highly patchy phenomena, such as bleaching, whereby the coral host expels their intracellular *Symbiodinium* spp., due to a combination of thermal and irradiance stress [77–79]. The occurrence of bleached and unbleached coral individuals of the same species found adjacent to one another may be explained by environmental heterogeneity [12] or due to selective mortality. This, however, is only conjecture as no phenotypic data were collected in this study.

Studying adaptation in the face of climate change has been difficult due to the uncertainty of how corals will respond to environmental differences. Conservation efforts have largely focused on maximizing population connectivity, however, connectivity refers not just to the transport of larvae but also their ability to recruit, survive, and create the next generation in their new home. In other words, habitat unsuitability can decrease levels of connectivity even in the face of considerable population mixing. This has been termed phenotype-environment mismatch [3, 80, 81] and could be a biological barrier to gene flow for organisms where the scale of environmental heterogeneity is smaller than the scale of larval transport and non-random mortality occurs after dispersal. Here, we show that this process may be occurring at the intra-reef scale as well. Predicting how species will respond to climate change, therefore, will require studying habitat suitability alongside genetic connectivity.

### A new paradigm for intra-reef coral genetic diversity

Marine genetic adaptive divergence can be seen as a continuum, with populations being pulled apart by selective forces to species whose reproductive isolation is maintained by ecological boundaries [82–84]. What we suggest here is a new paradigm for individual-level, intra-reef patterns of coral genetic diversity. Selection-driven genetic divergence has historically been viewed as difficult to occur if gene flow is high. Here, however, we demonstrate that despite genetic homogeneity on an inter-reef scale [13], genetic relatedness patterns within a reef are not random and instead, driven by both environmental and spatial factors. In other words, genetic differentiation may still arise among populations connected by high gene flow [84]. Unlike plant dispersal, marine larvae exhibit active microhabitat settlement choice and a renewed emphasis on the causes and consequences of larval retention has emerged [85]. In understanding the scale of gene flow in marine environments, therefore, one must move beyond purely spatial factors, and consider the influence of larval settlement behavior as well as post-recruitment selective mortality due to phenotype-environment mismatch.

It should be emphasized, however, that we only focus on inferring processes that can explain our observed depth cline in genetic diversity and multiple processes are likely co-occurring. Since most larval settlement studies have been conducted in the laboratory, it is still unknown how larvae in the field would respond to a suite of cues acting synergistically and on a variety of spatial scales [85, 31]. Just as population genetic studies attempt to infer processes of population connectivity, landscape genetic studies that focus on explaining processes of diversification within a continuous landscape may help to explain patterns of recruitment and larval behavioral responses to multiple cues acting simultaneously.

## Supporting Information

S1 Fig Spatial map of the lagged scores from the first three global principal components of sPCA based on spatial locations. Here, the three principal components are visualized simultaneously by translating each into a color (red, green, or blue) and displaying the combined mixed colors.
(TIF)

## References

[1] Mayr E. Systematics and the Origin of Species, From the Viewpoint of a Zoologist. New York, NY: Columbia University Press; 1942

[2] Hellberg ME. Gene flow and isolation among populations of marine animals. Ann Rev Ecol Evol Syst. 2009;40: 291–310.

[3] Conover DO, Clarke LM, Munch SB, Wagner GN. Spatial and temporal scales of adaptive divergence in marine fishes and the implications for conservation. J Fish Biol. 2006;69: 21–47.

[4] Rocha LA, Bowen BW. Speciation in coral-reef fishes. J Fish Biol. 2008;2: 1101–1121.

[5] Bird CE, Holland BS, Bowen BW, Toonen RJ. Diversification of sympatric broadcast-spawning limpets (Cellana spp.) within the Hawaiian archipelago. Mol Ecol. 2011;20: 2128–2141. 10.1111/j.1365-294X.2011.05081.x 21481050

[6] Sanford E, Kelly MW. Local adaptation in marine invertebrates. Ann Rev Mar Sci. 2011;3: 509–535. 21329215 10.1146/annurev-marine-120709-142756

[7] Bowen BW, Rocha LA, Toonen RJ, Karl SA, ToBo Laboratory. The origins of tropical marine biodiversity. TREE. 2013;359–366. 10.1016/j.tree.2013.01.018 23453048

[8] Sherman CDH, Ayre DJ (2008) Fine-scale adaptation in a clonal sea anemone. Evol. 2008;62: 1373–1380. 10.1111/j.1558-5646.2008.00375.x 18346218

[9] Barshis DJ, Stillman JH, Gates RD, Toonen RJ, Smith LW, Birkeland C. Protein expression and genetic structure of the coral Porites lobatain an environmentally extreme Samoan back reef: does host genotype limit phenotypic plasticity? Mol Ecol. 2010;19: 1705–1720. 10.1111/j.1365-294X.2010.04574.x 20345691

[10] Jørgensen HBH, Hansen MM, Bekkevold D, Ruzzante DE, Loeschcke V. Marine landscapes and population genetic structure of herring (Clupea harengus L.) in the Baltic Sea. Mol Ecol. 2005;14: 3219–3234. 16101787 10.1111/j.1365-294X.2005.02658.x

[11] Oetjen K, Reusch TB. Genome scans detect consistent divergent selection among subtidal vs. intertidal populations of the marine angiosperm Zostera marina. Mol Ecol. 2007;16: 5156–5157. 17986196 10.1111/j.1365-294X.2007.03577.x

[12] Gorospe KD, Karl SA. Small-scale spatial analysis of in situ sea temperature throughout a single coral patch reef. Journal of Mar Biol. 2011;2011: 12 pages 10.1155/2011/719580

[13] Gorospe KD, Karl SA. Genetic relatedness does not retain spatial pattern across multiple scales: dispersal and colonization in the coral, Pocillopora damicornis. Mol Ecol. 2013;22: 3721–3736. 10.1111/mec.12335 23786173

[14] Sotka EE. Local adaptation in host use among marine invertebrates. Ecol Let. 2005;8: 448–459.

[15] Kawecki TJ, Ebert D. Conceptual issues in local adaptation. Ecol Lett. 2004;7: 1225–1241.

[16] Vekemans X, Hardy OJ. New insights from fine-scale spatial genetic structure analyses in plant populations. Mol Ecol. 2004;13: 921–935. 15012766 10.1046/j.1365-294x.2004.02076.x

[17] Leimu R, Fischer M. A meta-analysis of local adaptation in plants. PLoS ONE, 2008;3: e4010 10.1371/journal.pone.0004010 19104660 PMC2602971

[18] Linhart YB, Grant MC. Evolutionary significance of local genetic differentiation in plants. Ann Rev Ecol Syst. 1996;27: 237–277.

[19] van Kleunen M, Fischer M. Adaptive evolution of plastic foraging responses in a clonal plant. Ecology. 2001;82: 3309–3319.

[20] Knight TM, Miller TE. Local adaptation within a population of Hydrocotyle bonariensis. Evol Ecol Res. 2004;6: 103–114.

[21] D’Croz L, Maté JL. Experimental responses to elevated water temperature in genotypes of the reef coral Pocillopora damicornis from upwelling and non-upwelling environments in Panama. Coral Reefs. 2004;23: 473–483.

[22] Vermeij MJA, Sandin SA, Samhouri JF. Local habitat distribution determines the relative frequency and interbreeding potential for two Caribbean coral morphospecies. Evol Ecol. 2007;21: 27–47.

[23] Storfer A, Murphy MA, Evans JS, Goldberg C, Robinson S, Spear S, et al Putting the “landscape” in landscape genetics. Hered. 2007;98: 128–142. 10.1038/sj.hdy.6800917 17080024

[24] Kalisz S, Nason JD, Hanzawa FM, Tonsor SJ. Spatial population genetic structure in Trillium grandiflorum: the roles of dispersal, mating, history, and selection. Evol. 2001;55: 1560–1568. 11580015 10.1111/j.0014-3820.2001.tb00675.x

[25] Toonen RJ, Pawlik JR. Foundations of gregariousness: a dispersal polymorphism among the planktonic larvae of a marine invertebrate. Evol. 2001;55: 2439–2454. 11831660 10.1111/j.0014-3820.2001.tb00759.x

[26] Baums IB. A restoration genetics guide for coral reef conservation. Mol Ecol. 2008;17: 2796–2811. 10.1111/j.1365-294X.2008.03787.x 18482262

[27] Meyer E, Davies S, Wang S, Willis BL, Abrego D, Juenger TE, et al Genetic variation in responses to a settlement cue and elevated temperature in the reef-building coral Acropora millepora. Mar Ecol Prog Ser. 2009;392: 81–92.

[28] Hurlbut CJ. The adaptive value of larval behavior of a colonial ascidian. Mar Biol. 1993;115: 253–262.

[29] Raimondi PT, Morse ANC. The consequences of complex larval behavior in coral. Ecology. 2000;81: 3193–3211.

[30] McEdward LR. The ecology of marine invertebrate larvae Boca Raton, FL, USA: CRC Press; 1995.

[31] Gleason DF, Hofmann DK. Coral larvae: from gametes to recruits. Jour Exper Mar Biol Ecol. 2011;408: 42–57.

[32] Goreau TF. The ecology of Jamaican coral reefs I. Species composition and zonation. Ecology. 1959;40: 67–90.

[33] Baird AH, Babcock RC, Mundy CP. Habitat selection by larvae influences the depth distribution of six common coral species. Mar Ecol Prog Ser. 2003;252: 289–293.

[34] McIntire EJB, Fajardo A. Beyond description: the active and effective way to infer processes from spatial patterns. Ecology. 2009;90: 46–56. 19294912 10.1890/07-2096.1

[35] Schoville SD, Bonin A, François O, Lobreaux S, Melodelima C, Manel S. Adaptive genetic variation on the landscape: methods and cases. Ann Rev Ecol Evol Syst. 2012;43: 23–43.

[36] Flot J-F, Tillier S. The mitochondrial genome of Pocillopora (Cnidaria: Scleractinia) contains two variable regions: the putative D-loop and a novel ORF of unknown function. Gene. 2007;401: 80–87. 17716831 10.1016/j.gene.2007.07.006

[37] Rasband, WS. ImageJ. U.S. National Institutes of Health. Research Service Branch. 1997; Available: http://imagej.nih.gov/ij/. Accessed 2015 Feb 24.

[38] Pebesma EJ, Wesseling CG. Gstat: a program for geostatistical modelling, prediction and simulation. Comp Geosci. 1998;24: 17–31.

[39] Hardy OJ, Vekemans X. Isolation by distance in a continuous population: reconciliation between spatial autocorrelation analysis and population genetics models. Hered. 1999;83: 145–154. 10.1046/j.1365-2540.1999.00558.x 10469202

[40] Hardy OJ, Vekemans X. Spagedi: a versatile computer program to analyse spatial genetic structure at the individual or population levels. Mol Ecol Not. 2002;2: 618–620.

[41] Mantel N. The detection of disease clustering and a generalized regression approach. Cancer Res. 1967;27: 209–220. 6018555

[42] Smouse PE, Long JC, Sokal RR. Multiple regression and correlation extensions of the Mantel test of matrix correspondence. Syst Zool. 1986;35: 627–632.

[43] Raufaste N, Rousset F. Are partial Mantel tests adequate? Evol. 2001;55: 1703–1705. 11580029 10.1111/j.0014-3820.2001.tb00689.x

[44] Castellano S, Balletto E. Is the partial Mantel test inadequate? Evol. 2002;56: 1871–1873. 10.1111/j.0014-3820.2002.tb00203.x 12389734

[45] Anderson MJ, Robinson J. Permutation tests for linear models. Aust New Zeal J Stat. 2001;43: 75–88.

[46] Legendre P, Fortin M-J. Comparison of the Mantel test and alternative approaches for detecting complex multivariate relationships in the spatial analysis of genetic data. Mol Ecol Res. 2010;10: 831–844. 10.1111/j.1755-0998.2010.02866.x 21565094

[47] Hughes TP, Connell JH. Population dynamics based on size or age? A reef-coral analysis. Am Nat. 1987;129: 818–829.

[48] Harriott VJ. Mortality rates of scleractinian corals before and during a mass bleaching event. Mar Ecol Prog Ser. 1985;21: 81–88.

[49] Excoffier L, Smouse PE, Quattro JM. Analysis of molecular variance inferred from metric distances among DNA haplotypes: application to human mitochondrial DNA restriction data. Genetics. 1992;131: 479–491. 1644282 10.1093/genetics/131.2.479 PMC1205020

[50] Meirmans P, van Tienderen P. Genotype and Genodive: two programs for the analysis of genetic diversity of asexual organisms. Mol Ecol Notes. 2004;4: 792–794.

[51] Burnham KP, Anderson DR. Kullback-Leibler information as a basis for strong inference in ecological studies. Wild Res. 2001;28: 111–119.

[52] Bartón K. MuMIn: Multi-model Inference, R package version 1.7.7. 2009;R–Forge website. Available: http://r-forge.r-project.org/projects/mumin. Accessed 2015 Feb 24.

[53] Akaike H. Information theory and an extension of the maximum likelihood principle In: Kotz S, Johnson NL, editors. Breakthroughs in Statistics. New York, NY: Springer; 1992 pp. 610–624.

[54] Anderson TJ, Haubold B, Williams JT, Estrada-Franco JG, Richardson L, Mollinedo R, et al Microsatellite markers reveal a spectrum of population structures in the malaria parasite Plasmodium falciparum. Mol Biol Evol. 2000;17: 1467–1482. 11018154 10.1093/oxfordjournals.molbev.a026247

[55] Jombart T, Devillard S, Dufour A-B, Pontier D. Revealing cryptic spatial patterns in genetic variability by a new multivariate method. Hered. 2008;101: 92–103. 10.1038/hdy.2008.34 18446182

[56] Jombart T. Adegenet: A R package for the multivariate analysis of genetic markers. Bioinfo. 2008;24:1403–1405. 10.1093/bioinformatics/btn129 18397895

[57] Thioulouse J, Chessel D, Champely S. Multivariate analysis of spatial patterns: a unified approach to local and global structures. Enviro Ecol Stat. 1995;2: 1–14.

[58] Griffith DA, Peres-Neto PR. Spatial modeling in ecology: The flexibility of eigenfunction spatial analyses. Ecology. 2006;87: 2603–2613. 17089668 10.1890/0012-9658(2006)87[2603:smietf]2.0.co;2

[59] Dray S, Legendre P, Peres-Neto PR. Spatial modelling: a comprehensive framework for principal coordinate analysis of neighbour matrices (PCNM). Ecol Model. 2006;196: 483–493.

[60] Johnson MS, Black R. Chaotic genetic patchiness in an intertidal limpet, Siphonaria sp. Mar Biol. 1982;70: 157–164.

[61] Johnson MS, Black R. Pattern beneath the chaos: the effect of recruitment on genetic patchiness in an intertidal limpet. Evol. 1984;38: 1371–1383. 10.1111/j.1558-5646.1984.tb05658.x 28563786

[62] Selkoe KA, Watson JR, White C, Horn TB, Iacchei M, Mitarai S, et al Taking the chaos out of genetic patchiness: seascape genetics reveals ecological and oceanographic drivers of genetic patterns in three temperate reef species. Mol Ecol. 2010;19: 3708–3726. 10.1111/j.1365-294X.2010.04658.x 20723063

[63] Toonen RJ, Grosberg RK. Causes of chaos: spatial and temporal genetic heterogeneity in the intertidal anomuran crab Petrolisthes cinctipes In: Koenemann S, Held C, Schubart C, editors. Phylogeography and Population Genetics in Crustacea. Boca Ratan, FL: CRC Press Crustacean Issues Series, 2011 pp.75–107.

[64] Carlon DB, Budd AF. Incipient speciation across a depth gradient in a scleractinian coral? Evol. 2002;56: 2227–2242. 10.1111/j.0014-3820.2002.tb00147.x 12487353

[65] Bongaerts P, Riginos C, Ridgway T, Sampayo EM, van Oppen MJH, Englebert N, et al Genetic divergence across habitats in the widespread coral Seriatopora hystrix and its associated Symbiodinium. PLoS ONE. 2010;5: e10871 10.1371/journal.pone.0010871 20523735 PMC2877717

[66] Costantini F, Rossi S, Pintus E, Cerrano C, Gili J-M, Abbiat M. Low connectivity and declining genetic variability along a depth gradient in Corallium rubrum populations. Coral Reefs. 2011;30: 991–1003.

[67] Richmond RH. Energetic relationships and biogeographical differences among fecundity, growth and reproduction in the reef coral Pocillopora damicornis. Bull Mar Sci. 1987;41: 594–604.

[68] Isomura N, Nishihira M. Size variation of planulae and its effect on the lifetime of planulae in three pocilloporid corals. Coral Reefs. 2001;20: 309–315.

[69] Tonsor SJ, Kalisz S, Fisher J, Holtsford TP. A life-history based study of population genetic structure: seed bank to adults in Plantago lanceolata. Evol. 1993;47: 833–843. 10.1111/j.1558-5646.1993.tb01237.x 28567912

[70] Stake JL, Sammarco PW. Effects of pressure on swimming behavior in planula larvae of the coral Porites astreoides (Cnidaria, Scleractinia). J Exp Mar Biol Ecol. 2003;288: 181–201.

[71] Smith-Keune C, Van Oppen M. Genetic structure of a reef-building coral from thermally distinct environments on the Great Barrier Reef. Coral Reefs. 2006;25: 493–502.

[72] Smith DB, Barshis D, Birkeland C. Phenotypic plasticity for skeletal growth, density and calcification of Porites lobata in response to habitat type. Coral Reefs. 2007;26559–567.

[73] Mundy CN, Babcock RC. Role of light intensity and spectral quality in coral settlement: Implications for depth-dependent settlement? J Exp Mar Biol Ecol. 1998;223: 235–255.

[74] Zepp RG, Shank GC, Stabenau E, Patterson KW, Cyterski M, Fisher W, et al Spatial and temporal variability of solar ultraviolet exposure of coral assemblages in the Florida Keys: importance of colored dissolved organic matter. Limn Oceano. 2008;53: 1909–1922.

[75] Dunne RP, Brown BE. Penetration of solar UVB radiation in shallow tropical waters and its potential biological effects on coral reefs; results from the central Indian Ocean and Andaman Sea. Mar Ecol Prog Ser. 1996;144: 109–118.

[76] Gleason DF, Wellington GM. Variation in UVB sensitivity of planula larvae of the coral Agaricia agaricites along a depth gradient. Mar Biol. 1995;123: 693–703.

[77] Brown BE. Coral bleaching: causes and consequences. Coral Reefs. 1997;16: S129–S138.

[78] Jones RJ, Hoegh-Guldberg O, Larkum AWD, Schreiber U. Temperature-induced bleaching of corals begins with impairment of the CO_2_ fixation mechanism in zooxanthellae. Plant Cell Enviro. 1998;21: 1219–1230.

[79] Warner ME, Fitt WK, Schmidt GW. Damage to photosystem II in symbiotic dinoflagellates: a determinant of coral bleaching. Proc Nat Acad Sci. 1999;96: 8007–8012. 10393938 10.1073/pnas.96.14.8007 PMC22178

[80] Nosil P, Vines TH, Funk DJ. Perspective: Reproductive isolation caused by natural selection against immigrants from divergent habitats. Evol. 2005;59: 705–719. 15926683

[81] Marshall DJ, Monro K, Bode M, Keough MJ, Swearer S. Phenotype-environment mismatches reduce connectivity in the sea. Ecol Let. 2010;13: 128–140. 19968695 10.1111/j.1461-0248.2009.01408.x

[82] Rocha LA, Robertson DR, Roman J, Bowen BW. Ecological speciation in tropical reef fishes, Proc Roy Soc B: Biol Sci. 2005;272: 573–579. 10.1098/2004.3005 PMC1564072 15817431

[83] Bird CE. Morphological and behavioral evidence for adaptive diversification of sympatric Hawaiian limpets (Cellana spp.). Integ Comp Biol. 2011;51: 466–473. 10.1093/icb/icr050 21700576

[84] Nosil P. Speciation with gene flow could be common. Mol Ecol. 2008;17: 2103–2106. 10.1111/j.1365-294X.2008.03715.x 18410295

[85] Levin LA. Recent progress in understanding larval dispersal: new directions and digressions. Integ Comp Biol. 2006;46: 282–297. 10.1093/icb/icj024 21672742

