# Supplementary figures and images for: Depth as an Organizing Force in Pocillopora damicornis: Intra-Reef Genetic Architecture
Source: PLoS One. 2015 Mar 25;10(3):e0122127. doi: 10.1371/journal.pone.0122127 (PMC4373699; doi:10.1371/journal.pone.0122127)

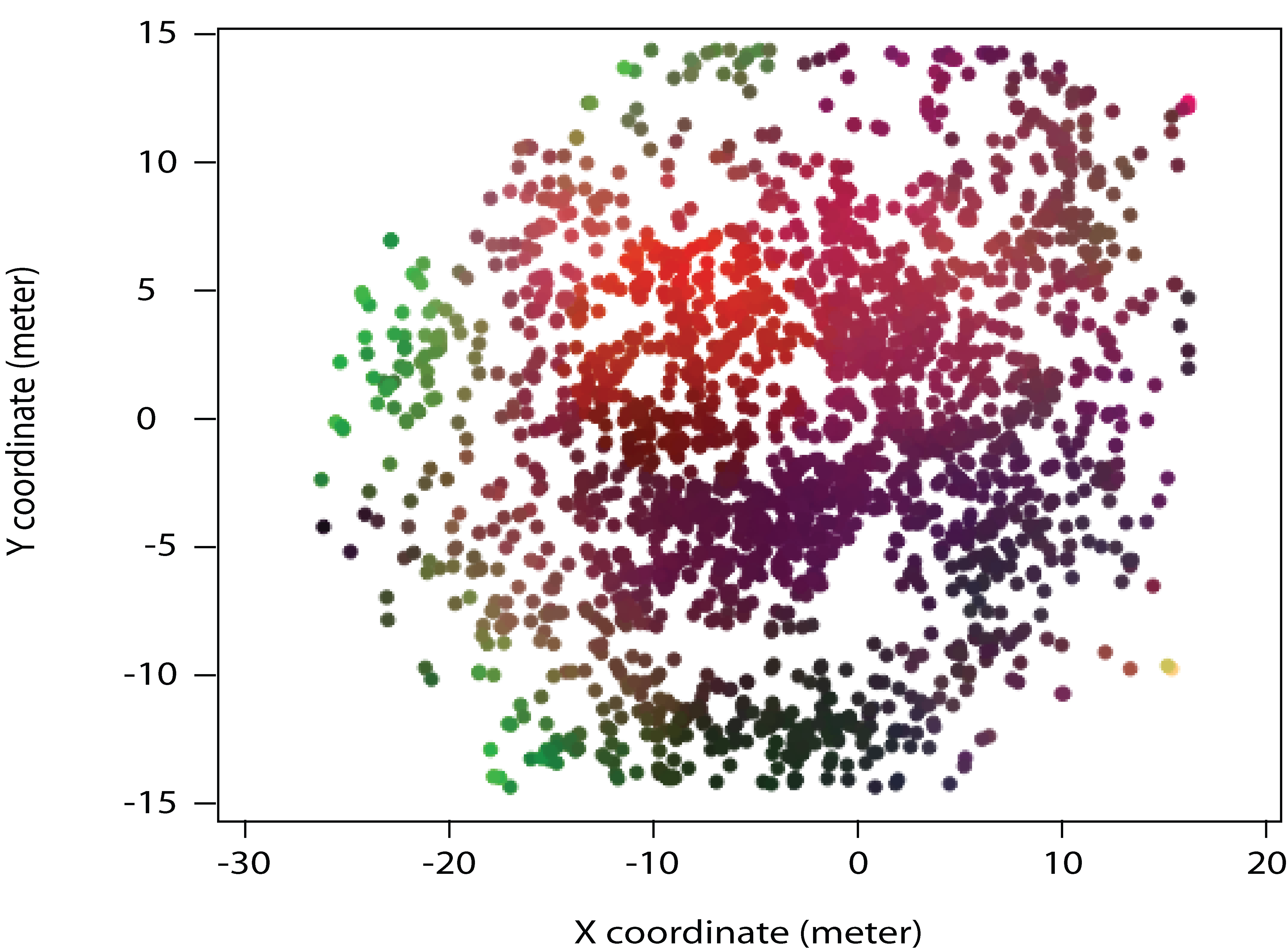

Supplement: S1 Fig — Here, the three principal components are visualized simultaneously by translating each into a color (red, green, or blue) and displaying the combined mixed colors. (TIF) [file pone.0122127.s001.tif]
